# Identification of a Novel ACE Inhibitory Hexapeptide from Camellia Seed Cake and Evaluation of Its Stability

**DOI:** 10.3390/foods12030501

**Published:** 2023-01-21

**Authors:** Qiaonan Zhu, Jiawen Xue, Peng Wang, Xianbo Wang, Jiaojiao Zhang, Xuezhi Fang, Zhiping He, Fenghua Wu

**Affiliations:** 1 College of Food and Health, Zhejiang Agriculture and Forestry University, Hangzhou 311300, China; 2 Zhejiang Feixiangyuan Food Co., Ltd., Lishui 323400, China; 3 Research Institute of Subtropical Forestry, Chinese Academy of Forestry, Hangzhou 311400, China; 4 College of Advanced Agricultural Sciences, Zhejiang Agriculture and Forestry University, Hangzhou 311300, China

**Keywords:** camellia seed cake, ACE inhibitory peptide, inhibitory mechanism, molecular docking, stability

## Abstract

The camellia seed cake proteins (CP) used in this study were individually hydrolyzed with neutral protease, alkaline protease, papain, and trypsin. The results showed that the hydrolysate had the highest ACE inhibitory activity at 67.36 ± 0.80% after four hours of neutral protease hydrolysis. Val-Val-Val-Pro-Gln-Asn (VVVPQN) was then obtained through ultrafiltration, Sephadex G-25 gel chromatography separation, LC-MS/MS analysis, and in silico screening. VVVPQN had ACE inhibitory activity with an IC_50_ value of 0.13 mg/mL (198.66 μmol/L), and it inhibited ACE in a non-competitive manner. The molecular docking indicated that VVVPQN can combine with ACE to form eight hydrogen bonds. The results of the stability study showed that VVVPQN maintained high ACE-inhibitory activity in weakly acidic and neutral environments and that heat treatment (20–80 °C) and Na^+^, Mg^2+^, as well as Fe^3+^ metal ions had little effect on the activity of VVVPQN. Moreover, it remained relatively stable after in vitro simulated gastrointestinal digestion. These results revealed that VVVPQN identified in camellia seed cake has the potential to be applied in functional food or antihypertensive drugs.

## 1. Introduction

Hypertension is a common chronic disease that can cause cardiovascular and cerebrovascular diseases, arteriosclerosis, and kidney diseases, among other conditions [1]. The prevalence of hypertension is gradually increasing as people’s living standards rise. Angiotensin I-converting enzyme (ACE) is a dipeptide carboxypeptidase that can convert inactive Angiotensin I (Ang I) to Angiotensin II (Ang II), which has the ability to contract blood vessels [2], causing elevated blood pressure. ACE has long been recognized as a key part of the renin angiotensin system that regulates blood pressure [3]. ACE inhibitors are important in the treatment of hypertension, which can effectively block the transformation of Ang I into Ang II, thus achieving the effect of treating hypertension [4]. Existing synthetic ACE inhibitors include benazepril, captopril, enalapril, etc. [5]. However, synthetic ACE inhibitors have some side effects, including taste disorders, coughing, skin rash, and angioedema [6]. When compared to chemically synthesized drugs, bioactive peptides derived from food proteins are more ideal as they offer superior health benefits and fewer side effects [7]. Therefore, developing natural ACE inhibitory peptides from foods that are beneficial to health and have few side effects has been a topic of considerable interest. After the first ACE inhibitory peptide was obtained from a snake, many ACE inhibitory peptides have been isolated from cereals, mushrooms, vegetables, beans, nuts, and their by-products [8]. Some technological processes used in food production, such as heating, acid or alkali treatment, and food ingredients (metal ions), may affect the functional and biological properties of peptides [9]. In addition, the bioactivity of the ACE inhibitory peptides can also be altered by gastrointestinal digestion [10]. Therefore, it is necessary to investigate the effects of food processing conditions and gastrointestinal digestion on the stability of ACE inhibitory peptides.

Camellia (*Camellia oleifera* Abel) is one of the four major woody oil crops (camellia oleifera, oil palm, olive, and coconut) in the world [11], mainly distributed in China, India, Japan, and Southeast Asian countries [12]. Camellia oil is rich in unsaturated fatty acids and phenolic compounds and has good nutritional value [13]. In China, about 2 million tons of camellia seed cake are produced annually, accounting for about 50% of camellia seed production [14]. Camellia seed cake is a by-product of oil extraction by pressing and it has a protein content of 10–20% [15], making it a potential protein source. However, currently, camellia seed cake is mainly used for traditional purposes such as animal feed, detergent, and organic fertilizer [16], and protein resources are not fully utilized. Plant-derived protein is the source of a variety of bioactive peptides, and it has been found that the peptides obtained after hydrolysis of camellia seed cake proteins (CP) have antioxidant, anti-tumor, hypoglycemic, hypotensive, and other activities [10], but there has been little research on the ACE inhibitory peptides of camellia seed cake.

In this study, CP were used as the raw material, and enzymatic hydrolysis was used to produce the hydrolysate of CP that had ACE inhibitory activity. The hydrolysate was further purified by ultrafiltration and Sephadex G-25 gel chromatography, and the amino acid sequence was analyzed by LC-MS/MS. The peptide with ACE inhibitory activity was obtained through in silico screening. Inhibition kinetics and molecular docking were both used to investigate its inhibition mode. In addition, the stability of the peptide at different temperatures, pH, metal ions, and gastrointestinal enzyme digestion was investigated in order to provide a theoretical basis for the development and utilization of ACE inhibitory peptides in camellia seed cake.

## 2. Materials and Methods

### 2.1. Materials

Camellia seeds were purchased from Quzhou City, Zhejiang Province. Angiotensin I-converting enzyme (ACE) from rabbit lungs (0.1 U) (Table A1), ACE substrate hippuryl-histidyl-leucine (HHL) and hippuric acid (HA) standards were purchased from Sigma-Aldrich (St. Louis, MO, USA). Neutral protease (50,000 U/g), alkaline protease (200,000 U/g), papain (800,000 U/g), trypsin (250,000 U/g), and Sephadex G-25 were purchased from Beijing Solarbio Science & Technology Co., Ltd. (Beijing, China). All other chemicals used were of analytical grade.

### 2.2. Preparation of CP

The preparation of CP was carried out by the method of Li [17] with some modifications. Camellia seed cake powder was mixed with petroleum ether at a ratio of 1:5 (*w*/*v*), stirred at room temperature for 2 h and the supernatant was discarded (repeat twice); the defatted powder was obtained after drying. Subsequently, the powder was mixed with 80% ethanol at a ratio of 1:10 (*w*/*v*) and stirred at 40 °C for 2 h; the supernatant was discarded (repeated twice). The defatted desaponin powder was obtained after drying. The powder was then dispersed in deionized water at a ratio of 1:20 (*w*/*v*, pH 10) and extracted by stirring at 50 °C for 2 h. The supernatant was collected by centrifugation and the pH was adjusted to 4.5. Following a 1-h standing period, the precipitate was centrifuged and washed with 80% ethanol (repeated three times). Finally, the precipitate was redissolved at pH 7 and lyophilized to obtain CP. The protein content of the CP was determined by the Kjeldahl method.

### 2.3. Preparation of CP Hydrolysate

To denature the protein, the CP (2%, *w*/*v*) was dispersed in deionized water and placed in a 95 °C water bath for 15 min. The pH of CP solution was adjusted with 1 mol/L NaOH or HCl to the optimal pH for each protease after cooling to room temperature. The solution was then treated with protease (5000 U/g) and hydrolyzed for 6 h. The optimum conditions for each protease are as follows: neutral protease (45 °C, pH 7), alkaline protease (50 °C, pH 10), papain (40 °C, pH 6), trypsin (37 °C, pH 8). Every hour, a portion of the solution was taken out, placed in 95 °C water for 15 min to inactivate the enzymes, cooled to room temperature, and then centrifuged at 8500 rpm for 30 min. The degrees of hydrolysis (DH) and ACE inhibitory activity were measured, respectively. The protease, the hydrolysate of which had the highest DH and ACE inhibitory activity, was selected as the optimal protease, and its hydrolysate was collected as CPH.

### 2.4. Degree of Hydrolysis

The DH was determined by the o-phthaldehyde (OPA) method, which is based on the reaction of free amino groups with OPA to form a yellow complex [18]. The absorbance at 340 nm was determined after a reaction of 2 min between 400 µL of the enzymatic solution and 3 mL of the OPA reagent. The DH was calculated using the following formula:

Serine NH_2_ (L/g) = [(OD_sample_ − OD_blank_)/(OD_standard_ − OD_blank_)] × 0.9516 × [(0.1 × 100)/(X × P)](1)

h (mmol/g) = (Serine NH_2_ − β)/α (2)

DH (%) = (h/h_tot_) × 100% (3)

where OD_sample_ is the absorbance of the sample solution, OD_blank_ is the absorbance of the equal volume of water instead of the sample, OD_standard_ is the absorbance of the equal volume of the serine solution at 340 nm; 0.9516 is the molar concentration of the serine standard solution.; X is the sample weight in grams (g); P is the CP’s protein content in percentage (78.58%); 0.1 is the sample volume in liters (L); and α, β, h_tot_ is entirely dependent on the type of raw material (α: 1.00, β: 0.40, h_tot_: 8).

### 2.5. ACE Inhibitory Activity

The ACE inhibition rate was determined using High-Performance Liquid Chromatography (HPLC) [19]. In summary, 60 μL of HHL (2.5 mM) and 20 μL of samples were mixed and incubated at 37 °C for 5 min before 40 μL of ACE (0.05 U/mL) was added. The mixture was incubated at 37 °C for 1 h. Finally, the reaction was terminated by adding 120 μL of HCl solution (1 mol/L). The content of HA in the reaction system was detected by a PITC pre-column derivatization HPLC (Shimadzu LC-20AT) with a column C18 Hypersil ODS2 (4.6 mm×250 mm, 5 μm) at 228 nm. Isocratic elution was processed with the acetonitrile-water (volume ratio 1:3, each containing 0.1% TFA) solution at a constant flow rate of 1 mL/min. The ACE inhibition rate (%) was calculated based on the following formula:

ACE inhibition rate (%) = [(A_0_ − A_1_) / A_0_] × 100% (4)

where A_0_ and A_1_ equal the HA peak areas of the blank control and sample, respectively.

### 2.6. Ultrafiltration

The CPH solution was fractionated using an ultrafiltration system (Millipore, Bedford, MA, USA) with molecular weight (MW) cutoffs of 3 kDa and 10 kDa. Three fractions were obtained: CPH1: > 10 kDa, CPH2: 3–10 kDa, and CPH3: < 3 kDa. The ACE inhibitory activity of all fractions was determined as described in Section 2.5.

### 2.7. Purification by Sephadex G-25

The active fraction was further purified using a Sephadex G-25 gel column (Φ16 × 700 mm). The sample solution at a concentration of 10 mg/mL was filtered through a 0.45 μm membrane filter. The filtrate was collected and separated on a Sephadex G-25 gel column eluted by deionized water with an elution rate of 0.8 mL/min and monitored at 280 nm.

### 2.8. Peptide Identification by LC-MS/MS

The peptide sequence was determined by liquid chromatography coupled with tandem mass spectrometry (LC-MS/MS) at the Beijing Biotech-Pack Scientific Co., Ltd. The mass spectrometer used was a Q Exactive™ Hybrid Quadrupole-Orbitrap™ (Thermo Fisher Scientific, Waltham, MA, USA). The peptide sample was reduced by 10 mM DTT at 56 °C for 1 h, alkylated by 50 mM IAA at room temperature in the dark for 40 min, and lyophilized to dryness. Prior to LC-MS/MS analysis, the peptide was resuspended in 20 μL of 0.1% formic acid. Data analysis was performed using PEAKS Studio (8.5) De novo software.

### 2.9. In Silico Screening and Synthesis of Peptides

Only peptides with a De novo score higher than 95 and an area higher than 10^8^ were selected to be further processed for molecular docking analysis. Peptides with potential ACE inhibitory activity were screened according to binding energy. The pI and GRAVY were determined using a Thermo Fisher Peptide analyzing tool server (https://www.thermofisher.cn/cn/zh/home/life-science/protein-biology/peptides-proteins/custom-peptide-synthesis-services/peptide-analyzing-tool.html accessed on 29 September 2022). In order to verify the ACE inhibitory activity, the selected peptides were synthesized by GenScript (Nanjing, China). The MW and purity of the synthetic peptides were determined by MS and HPLC, respectively.

### 2.10. Molecular Docking

Molecular docking was performed with AutoDock Vina software. The 3D structure of ACE (PDB: 1O8A) was derived from the Protein Data Bank. The 3D structure of peptides was generated by ChemBioDraw Ultra 14.0 and the energy minimized by ChemBio3D Ultra 14.0 software (Cambridge Soft, Cambridge, MA, USA). All hetero molecules in 1O8A, including water, were removed prior to docking, while the cofactors zinc and chloride ions were retained. The optimal binding pose for a peptide with ACE was predicted based on binding affinity. The 3D-structure diagrams showing the interaction between the peptide and ACE were drawn with PyMol 1.7 software.

### 2.11. Inhibitory Pattern of VVVPQN

The ACE inhibition pattern of the VVVPQN was determined in accordance with the method reported by Urbizo-Reyes [20], with some modifications. Peptide samples (0, 0.05, and 0.20 mg/mL) and various concentrations of HHL (2, 3, 4, and 5 mmol/L) were mixed with ACE according to the method described in Section 2.5. A Lineweaver–Burk plot was used to analyze the inhibitory pattern, which was based on the reciprocal of the HA production rate (1/v) and the HHL concentration. (1/[s]).

### 2.12. Stability of VVVPQN

#### 2.12.1. Temperature

The 0.4 mg/mL VVVPQN solution was placed in a water bath at varying temperatures (20, 40, 60, 80, and 100 °C) for 2 h. After cooling to room temperature, the ACE inhibition rate of VVVPQN was determined using the method described in Section 2.5.

#### 2.12.2. pH

The 0.4 mg/mL VVVPQN solution was adjusted to varying pH values (3, 5, 7, 9, and 11) with HCl (1 mol/L) or NaOH (1 mol/L). After incubation at 37 °C for 2 h, the pH was adjusted to 7.0 before the ACE inhibition rate was measured.

#### 2.12.3. Metal Ions

VVVPQN was dissolved in distilled water (10 mg/mL), and diluted to 0.4 mg/mL with K^+^, Na^2+^, Ca^2+^, Mg^2+^, and Fe^3+^ solutions at concentrations of 0, 2, 4, 6, and 8 mmol/L, respectively. The mixture was incubated at 37 °C for 2 h, and 20 μL of the mixture was sucked out and used to measure the ACE inhibition rate.

#### 2.12.4. Stability after In Vitro Treatment with Digestive Enzymes

The stability of VVVPQN after in vitro treatment with digestive enzymes was carried out by the method of Chai [21] with some modifications. Briefly, in a water bath at 37 °C, VVVPQN (0.4 mg/mL) was first digested with 2% (*w*/*w*) pepsin at pH 2.0 for 2 h. The pH was then adjusted to 7.0 before trypsin was added at a 1:50 enzyme to substrate ratio. After digestion for 2 h at 37 °C, the enzyme was inactivated by heating for 10 min at 95 °C. The stability of VVVPQN was evaluated by comparing the ACE inhibition rate before and after the digestion.

### 2.13. Statistical Analysis

All the experiments were performed in triplicates, and the results were expressed as mean ± SD (n = 3). The SPSS 23.0 software (SPSS Inc., Chicago, IL, USA) was used to perform a one-way analysis of variance (ANOVA) on the data in this study. Significant differences were considered when *p* < 0.05.

## 3. Results and Discussion

### 3.1. Preparation and Purification of CPH

#### 3.1.1. Preparation of CPH

**Figure 1:**
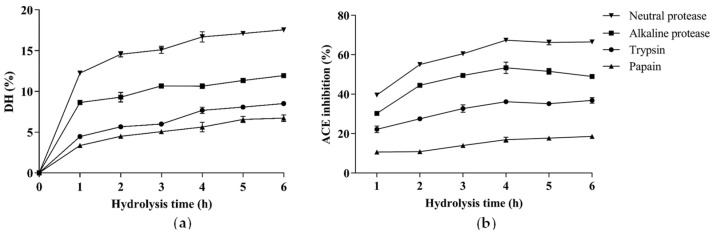
The degree of hydrolysis (**a**) and ACE inhibitory activity (**b**) of hydrolysates with alkaline protease, papain, neutral protease, and trypsin. The results are presented as the means ± SD (n = 3).

The effects of protease species on the DH and ACE inhibitory activities of hydrolysates of CP are shown in Figure 1. Apparently, among the hydrolysates of the four proteases, the hydrolysate of the neutral protease showed the greatest DH and ACE inhibitory activity. The neutral protease hydrolysate exhibited the greatest ACE inhibitory activity when the hydrolysis duration was 4 h (DH = 16.69 ± 0.63%), reaching 67.36 ± 0.80% (1 mg/mL), which was significantly higher than other hydrolysates. Previous studies have shown that neutral protease is an endopeptidase without specific restriction sites, so it can produce lower molecular weight peptides [22], which tend to exhibit greater ACE inhibitory activity than high molecular weight peptides [23]. Therefore, the neutral protease’s hydrolysate released after 4 h (CPH) was further purified.

#### 3.1.2. Ultrafiltration

**Figure 2:**
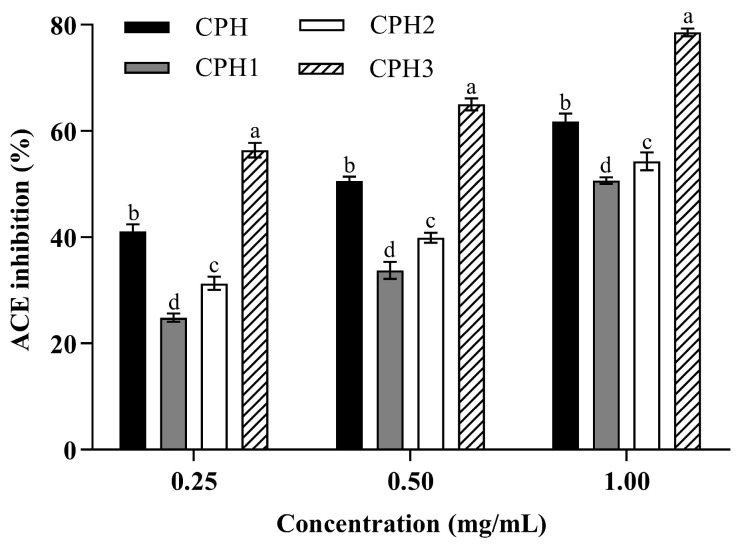
ACE inhibitory activity of CPH and its three ultrafiltration fractions (CPH1 to CPH3). The results are presented as the means ± SD (n = 3). Different letters indicate significant differences at *p* < 0.05.

Ultrafiltration was commonly used to separate peptides of different molecular weights. In general, peptides with a lower molecular weight showed more significant ACE inhibitory activity [24]. CPH was fractionated by ultrafiltration membranes with MWCO of 10 kDa and 3 kDa, and three peptide fractions were obtained, including CPH1, CPH2, and CPH3. The ACE inhibitory activity of CPH and the three fractions was measured at the concentrations of 0.25, 0.5 and 1.0 mg/mL. As shown in Figure 2, it was clear that the ACE inhibitory activity of each fraction increased with the increase in concentration. The CPH3 showed the highest ACE inhibitory activity, reaching 78.56% at a concentration of 1 mg/mL, which was significantly higher than the fractions before ultrafiltration (CPH) and the other two fractions. These results were consistent with the study reported previously [19], where the <3 kDa fraction of soybean protein isolate hydrolysate (SPIH) showed the greatest ACE inhibitory activity compared to the 3–5 kDa, 5–10 kDa and >10 kDa.

#### 3.1.3. Sephadex G-25 Gel Chromatography

**Figure 3:**
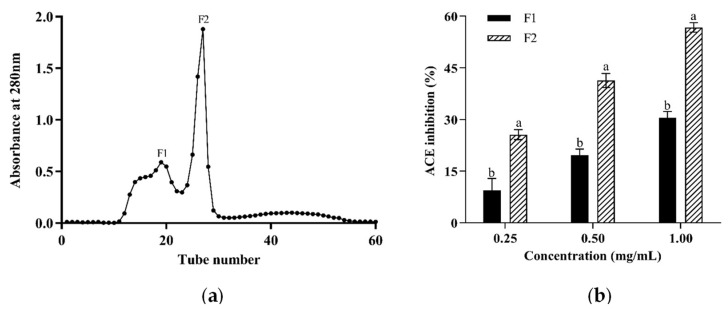
Sephadex G-25 gel chromatography and ACE inhibitory activity of subfractions (F1, F2) from CPH3. (**a**) Sephadex G-25 gel chromatography. (**b**) ACE inhibitory activity. The results are presented as the means ± SD (n = 3). Different letters indicate significant differences at *p* < 0.05.

When CPH3 was purified by a Sephadex G-25 column, there were two major absorbance peaks (Figure 3a) at 280 nm, and two fractions (F1, F2) associated with the peaks were collected and lyophilized. The ACE inhibitory activity of each fraction was determined at the concentrations of 0.25, 0.5 and 1.0 mg/mL, as shown in Figure 3b. It was clear that fraction F2 showed stronger ACE inhibitory activity compared to fraction F1. Consequently, LC-MS/MS was applied to identify the amino acid sequence of fraction F2.

### 3.2. Identification of Peptides from F2 and In Silico Screening

**Figure 4:**
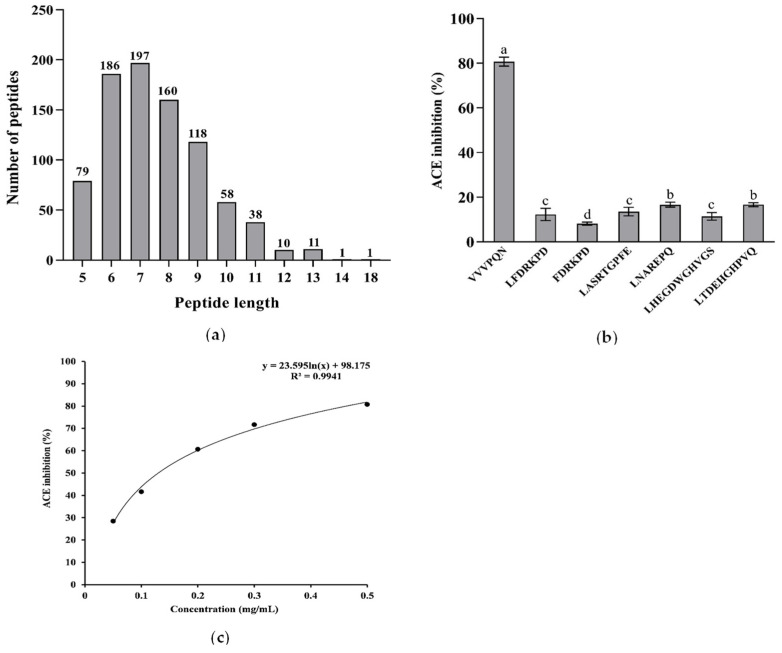
(**a**) The number of peptides with varying lengths identified from the F2 component. (**b**) The selected peptides’ ACE inhibitory activity. (**c**) The ACE inhibitory activity and the regression analysis of VVVPQN. The results are presented as the means ± SD (n = 3). Different letters indicate significant differences at *p* < 0.05.

**Figure 5:**
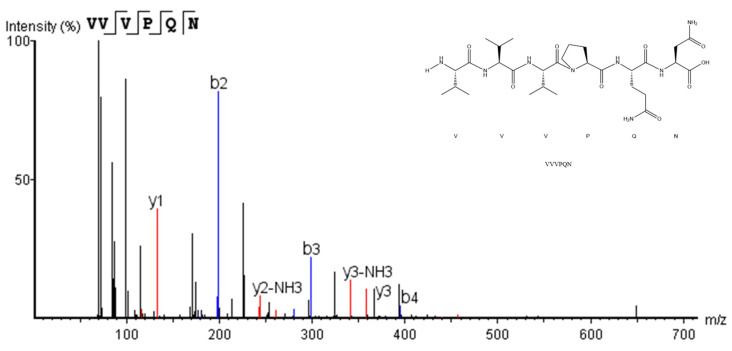
The MS/MS spectrum of the peptide VVVPQN.

ACE inhibitory peptides are usually short-chain peptides containing 2–12 amino acids [25]. A total of 859 peptides were identified in this study. The identified peptides were comprised of 5–18 amino acids, with 6–9 amino acids constituting the majority (Figure 4a). Peptides with the following characteristics: A de-novo score higher than 95, a peak area higher than 10^8^, and a binding energy below −7.5 kcal/mol were selected, including seven peptides (VVVPQN, LFDRKPD, FDRKPD, LASRTGPFE, LNAREPQ, LHEGDWGHVGS, and LTDEHGHPVQ). Some physicochemical properties, such as pI and GRAVY, of the seven peptides were shown in Table 1. They were then chemically synthesized with a purity above 95% and their ACE inhibitory activity was verified, respectively. As shown in Figure 4b, VVVPQN, the highest content peptide in F2 component (6.47%), showed the best ACE inhibitory activity as well, reaching 80.46% at a concentration of 0.5 mg/mL. Hence, VVVPQN was considered to contribute more than other components in F2 to the ACE inhibitory. Moreover, it was a novel ACE inhibitory peptide that had not been reported previously. Its MS/MS spectrum is shown in Figure 5. Based on the regression equation (y = 23.595 ln(x) + 98.175) as shown in Figure 4c, the IC_50_ value of the ACE inhibitory activity of VVVPQN was 0.13 mg/mL (198.66 μmol/L), which was lower than GYGYNY’s from camellia glutelin-2 hydrolysates (IC_50_: 384 μmol/L) [10]. The ratio of hydrophobic to hydrophilic amino acids could affect peptides’ ACE inhibitory activity according to previous studies [25]. The content of hydrophobic amino acids in VVVPQN was 66.67%, which was significantly higher than that of the other six peptides identified in this study. In addition, some peptides that have been identified in previous studies, such as peptides VVNE, VVTR, and VVGVD derived from wild almond proteins [26], peptide VTPVGVPKW isolated from black cumin seed [27], and peptide VPAAPPK derived from haruan (*Channa striatus*) myofibrillar protein hydrolysate, have indicated that peptides with Val at the N-terminal have high ACE inhibitory activity [28]. This may explain the potent ACE inhibitory activity of VVVPQN.

### 3.3. Inhibitory Pattern of VVVPQN

**Figure 6:**
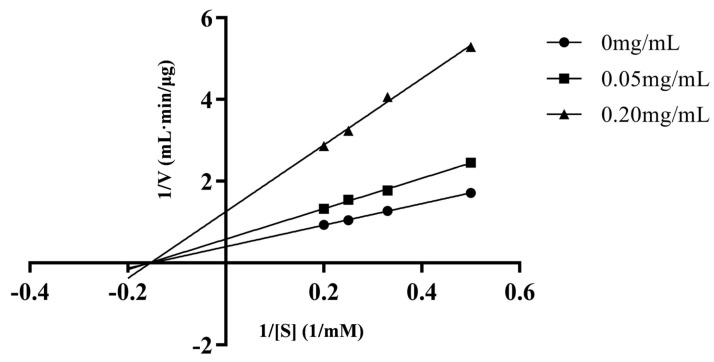
Lineweaver–Burk plots for the ACE inhibition pattern of VVVPQN. 1/[S] and 1/V represent the reciprocal of substrate concentration and reaction velocity, respectively.

The inhibition pattern of VVVPQN was analyzed by a Lineweaver–Burk plot with four concentrations of substrate (2, 3, 4, and 5 mmol·L^−1^ HHL) and three concentrations of VVVPQN (0, 0.05, and 0.20 mg·mL^−1^). It can be seen in Figure 6 and Table 2 that when VVVPQN was added, Vmax decreased with the increase in peptide concentration, whereas Km did not change (6.67 mM), indicating that VVVPQN was a non-competitive inhibitor. Many ACE inhibitory peptides identified from food origins have been found to act in a non-competitive manner, such as peptides ADVFNPR and VIEPR isolated from oil palm kernel [29,30], peptide QLDL derived from the Mycelia of *Ganoderma Lucidum* [31], peptides GVSLPEW, GYGGVSLPEW and VGINYW derived from α-lactalbumin [32]. Non-competitive inhibitors can bind to the allosteric site of the enzyme or combine with the ACE molecule to produce a dead-end complex [33], regardless of whether the enzyme is bound to a substrate, resulting in conformational changes that reduce enzyme activity or prevent product formation [27]. Therefore, a complex can be formed between VVVPQN and ACE or the substrate to prevent the formation of the reaction product HA.

### 3.4. Molecular Docking Simulation between ACE and VVVPQN

**Figure 7:**
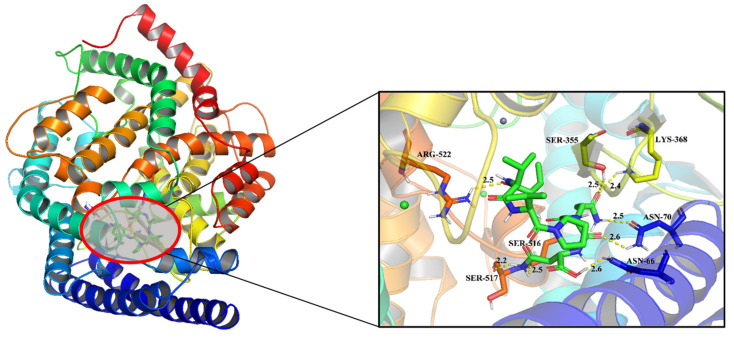
Molecular docking between ACE (PDB: 1O8A) and VVVPQN.

To further explore the combination pattern between ACE and VVVPQN, the docking simulation was analyzed using PyMol software. Previous studies indicated that ligands and receptors interact with each other through different intermolecular forces, including hydrophobic, hydrogen bonds, π bonds, electrostatic interaction, and Van der Waals’s force. Among them, the interaction force of hydrogen bonds plays the most important role [34]. The main active site of ACE consists of three active pockets (S1, S2, and S1′) [35]. The S1 pocket contains ALA354, GLU384 and TYR523, while the S2 pocket contains GLN281, HIS353, LYS511, HIS513, and TYR520, and the S1′ pocket only contains GLU162 [36]. The molecular docking models and results are shown in Figure 7 and Table 3, respectively. VVVPQN can form eight hydrogen bonds with ACE active sites, including SER516, SER517, ARG522, SER355, LYS368, ASN66, and two hydrogen bonds with ASN70. It is well known that noncompetitive inhibitors can combine with ACE at a site other than the active sites in active pockets, thereby inactivating the entire complex [25]. The results of molecular docking showed that the seven binding sites between the non-competitive inhibitory peptide VVVPQN and ACE were not within the three active pockets (S1, S2, and S1′), which was consistent with the results of inhibition kinetics.

### 3.5. The Stability of VVVPQN

#### 3.5.1. Thermal and pH Stability

**Figure 8:**
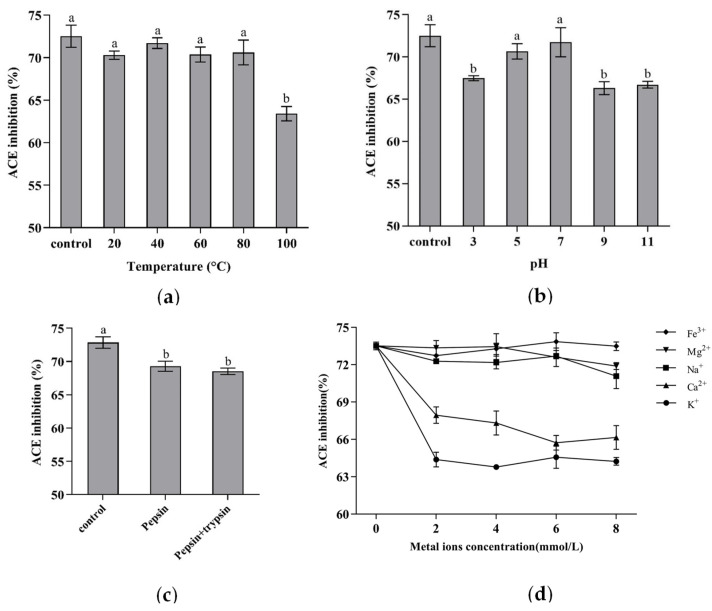
Stability of VVVPQN under heat treatment (**a**), different pH environments (**b**), gastrointestinal digestion (**c**), and addition of metal ions (**d**). The results are presented as the means ± SD (n = 3). Different letters indicate significant differences at *p* < 0.05.

The thermal and pH stability of peptides is an important factor of concern in peptide production and processing [37]. According to previous studies, high temperatures may alter the peptide’s secondary structure and sequence, thus affecting the ACE inhibitory activity [38]. As shown in Figure 8a, when the temperature was raised from 20 °C to 80 °C, the ACE inhibitory activity of VVVPQN did not decrease significantly and remained nearly intact. However, when the temperature was further increased to 100 °C, the ACE inhibitory activity of VVVPQN was significantly reduced. When compared to the control group, VVVPQN’s ACE inhibitory activity dropped by 9.11% at 100 °C. The pH stability research results of VVVPQN are shown in Figure 8b; the ACE inhibitory activity of VVVPQN did not change significantly at the pH values of 5 and 7, but decreased significantly at the pH values of 3, 9, and 11. This may be due to the fact that strong acids or alkalis can induce degradation, and change in the pH can affect the ionization state of peptides [10]. Especially in alkaline environments, ACE inhibitory peptides can undergo racemization and deamidation or further hydrolyze to form other substances without ACE inhibitory activity [39], thus changing the structure of the peptide and affecting its activity. As a result, it is best to process and utilize VVVPQN in a neutral environment.

#### 3.5.2. Stability after In Vitro Treatment with Digestive Enzymes

The ACE inhibitory peptide can exert a blood pressure-lowering effect in vivo if it reaches the blood stream in active form. Therefore, oral peptides need to resist hydrolysis by pepsin and trypsin while maintaining their bioactivity [40]. The effect of in vitro gastrointestinal simulated digestion on the ACE inhibitory activity of VVVPQN can be seen in Figure 8c. The activity of VVVPQN decreased significantly after simulated gastric digestion with pepsin in vitro, but the activity of VVVPQN did not change significantly when the intestinal digestion was further simulated with trypsin. These results may be related to the pH environment; the optimal pH of pepsin is acidic, while the optimal pH of trypsin is neutral. From studies on the stability of VVVPQN at different pH values, it can be concluded that the ACE inhibitory activity of VVVPQN decreases in the strong acid environment but hardly changes in the neutral environment. It is important to emphasize that although the ACE inhibitory activity of VVVPQN was reduced after pepsin treatment, it still maintained a high activity. Therefore, it can be assumed that VVVPQN had good anti-digestibility capacity. Previous studies have shown that small peptides containing between two and six amino acids contain fewer enzyme-sensitive peptide bonds and are less structurally flexible. Therefore, they are less susceptible to digestive enzymes [25].

#### 3.5.3. Metal Ion Stability

Metal ions were found in food-processing systems as a result of added salt and water or used metal containers [10]. The presence of these metal ions may affect the properties and biological activities of polypeptides. The effect of adding metal ions on the ACE inhibitory activity of VVVPQN is shown in Figure 8d. The addition of Na^+^, Mg^2+^, and Fe^3+^ ions had almost no effect on the ACE inhibitory activity of VVVPQN. However, the ACE inhibitory activity of VVVPQN decreased significantly after the addition of K^+^ and Ca^2+^. These results were not consistent with those obtained by [10,39]. Despite the fact that HPVTGL, GYGYNY, and VVVPQN are all hexapeptides, their activities may be affected differently by metal ions due to their different spatial structures.

## 4. Conclusions

The purified and identified novel ACE inhibitory peptide (VVVPQN) from CP exhibited an IC_50_ of 0.13 mg/mL (198.66 μmol/L) and a noncompetitive inhibition mode. Molecular docking showed that VVVPQN could form eight hydrogen bonds with ACE. In addition, VVVPQN had good thermal stability (20–80 °C) and it remained stable under neutral and weak acid conditions. The addition of Na^+^, Mg^2+^, and Fe^3+^ had no significant effect on ACE inhibitory activity and showed satisfactory residual activity after simulating gastrointestinal digestion conditions in vitro. Therefore, it can be assumed that VVVPQN has a potential application prospect as a functional additive in food or health products.

## Figures and Tables

**Table 1:** Binding energy and physicochemical characteristics of the selected peptides.

Peptide	Mass (Da)	Composition (%)	Score	Binding Energy (kcal/mol)	pI	GRAVY	Content of HAA (%)
VVVPQN	654.4	6.47%	97	−7.8	6.0	0.67	66.67%
LFDRKPD	889.5	3.54%	99	−8.2	6.9	−1.49	50.00%
FDRKPD	776.4	1.27%	99	−8.8	6.9	−2.37	33.33%
LASRTGPFE	976.5	1.27%	97	−9.0	7.0	−0.34	55.56%
LNAREPQ	826.4	1.12%	96	−8.9	7.0	−1.57	42.86%
LHEGDWGHVGS	1192.5	3.08%	95	−9.4	5.0	−0.75	45.45%
LTDEHGHPVQ	1131.5	2.08%	99	−9.9	5.0	−1.16	40.00%

pI: isoelectric point; GRAVY: Grand average of hydropathicity; HAA: hydrophobic amino acids.

**Table 2:** Kinetic parameters of VVVPQN binding with ACE in different concentrations.

VVVPQN	0 mg/mL	0.05 mg/mL	0.20 mg/mL
Vmax (μg/mL·min) Km (mM)	2.52 6.67	1.72 6.67	0.80 6.67

**Table 3:** Interactions between ACE and VVVPQN from the molecular docking simulation.

Peptides	Hydrogen Bonds Number	Interacting Residues	Distance (Å)
VVVPQN	8	SER516	2.5
		SER517	2.2
		ARG522	2.5
		SER355	2.5
		LYS368	2.4
		ASN66	2.6
		ASN70	2.5, 2.6

## Data Availability

Not applicable.

## Appendix A

**Table A1:** Product information and EC numbers of all the enzymes used in this paper.

Enzyme	CAS Number	Product Code	EC Number
Angiotensin I-converting enzyme	9015-82-1	A6778	EC 3.4.15.1
Neutral protease	9068-59-1	Z8031	EC 3.4.24.4
Alkaline protease	9014-01-1	B8360	EC 3.4.21.14
Papain	9001-73-4	G8432	EC 3.4.22.2
Trypsin	9002-07-7	T8150	EC 3.4.21.4
Pepsin	9001-75-6	P8160	EC 3.4.23.1
